# An analysis of the associations of high-sensitivity C-reactive protein and uric acid with metabolic syndrome components in Korean adults by sex: a cross-sectional study using the Korea national health and nutrition examination survey 2016–2018

**DOI:** 10.1186/s12902-023-01417-z

**Published:** 2023-08-03

**Authors:** Young Kyun Kim, Young-Mo Yang

**Affiliations:** Department of Pharmacy, College of Pharmacy, Chosun University, 309 Pilmun-daero, Dong-gu, Gwangju, 61452 Republic of Korea

**Keywords:** High-sensitivity C-reactive protein, Uric acid, Metabolic syndrome, Sex, Koreans, Cross-sectional study

## Abstract

**Background:**

Low-grade inflammation plays a role in the pathogenesis of metabolic syndrome (MetS), and measuring levels of inflammatory molecules, such as high-sensitivity C-reactive protein (hs-CRP), may indicate Mets progression. Serum uric acid (SUA) has also been identified as an independent risk factor for MetS. This study aimed to investigate the association between MetS components and levels of serum hs-CRP and SUA using representative and reliable data for the Korean population.

**Methods:**

This study used the data of the Korea National Health and Nutrition Examination Survey 2016–2018, a cross-sectional and nationally representative survey performed by the Korean Centers for Disease Control and Prevention.

**Results:**

We analysed the data of 13,454 individuals. High hs-CRP levels were observed in 1,164 (8.7%) subjects while 3,296 (24.5%) subjects had high SUA levels. Moreover, hs-CRP was negatively correlated with serum high-density lipoprotein (HDL) (OR, 1.703; 95% CI, 1.431–2.027). When stratified by sex, this trend remained, but the correlation was stronger in women than in men. Furthermore, high SUA levels were significantly associated with hypertension (HTN) (OR, 1.399; 95% CI, 1.210–1.616), hypertriglyceridemia (OR, 1.735; 95% CI, 1.486–2.026), and low HDL (OR, 1.257; 95% CI, 1.106–1.429), but not with diabetes mellitus (DM) (OR, 0.478; 95% CI, 0.382–0.597). When grouped by sex, this trend remained, however, all MetS components were found to be more prevalent in women with high SUA.

**Conclusions:**

Our findings showed that low HDL was more prevalent in subjects with high hs-CRP, and high SUA levels were observed in subjects with HTN, hypertriglyceridemia, and low HDL. However, the prevalence of high SUA was lower in diabetic subjects.

## Background

Metabolic syndrome (MetS) is a cluster of metabolic abnormalities that include abdominal obesity with high waist circumference (WC), increased blood pressure (BP), increased fasting blood glucose (FBG) and triglyceride (TG) levels, and decreased high-density lipoprotein (HDL) cholesterol levels [1–3]. These abnormalities are the well-known classical risk factors for type 2 diabetes mellitus (DM) and cardiovascular diseases (CVDs) [2, 3]. Worldwide, the prevalence of MetS has rapidly increased over the past several decades, and it is estimated that about one-quarter of adults have MetS, although its prevalence differs in different geographical regions [4–9]. Thus, it is crucial to identify early risk factors for MetS that could ultimately help in preventing abnormal conditions leading to chronic diseases.

Previous studies suggest that MetS is a chronic condition with low-grade inflammation and is influenced by the interaction between genetic and environmental factors [10, 11]. Although the precise role of inflammation in MetS pathogenesis remains unknown, the rate of disease progression could be indirectly estimated by measuring the circulating levels of inflammatory molecules such as high-sensitivity C-reactive protein (hs-CRP) [10, 11]. Mirhafez et al. reported that serum hs-CRP level increases in a step-wise manner with the increase in the number of MetS components [1]. Similar association was observed in another study conducted by Wang et al. [12].

Increased level of serum uric acid (SUA), the end product of purine metabolism, is considered as one of the independent risk factors for MetS [13–15]. High SUA levels might contribute to the development of MetS by enhancing the levels of circulatory inflammatory molecules, such as hs-CRP, and increasing the erythrocyte sedimentation rate by activating nuclear factor kappa-light-chain-enhancer of activated B cells (NF-kB), a protein complex that plays a role in the transcription of several cytokines and inflammatory molecules [14]. Thus, it is possible to predict the development of hypertension, obesity, and DM by monitoring the levels of serum hs-CRP and SUA.

The independent risk factors of hs-CRP and SUA levels may have a significant impact on evaluating the prevalence of MetS components in both men and women. Moreover, research has shown that there is a correlation between BMI and the increase in SUA and hs-CRP levels in adults [13, 16]. Nevertheless, there have been limited investigations into these relationships among Korean adults. Hence, the aim of this study was to explore the associations between the levels of hs-CRP and SUA and the prevalence of MetS components among Korean adults, categorized by sex and BMI subgroups, using representative and reliable data for the Korean population.

## Methods

### Study population

**Fig. 1:**
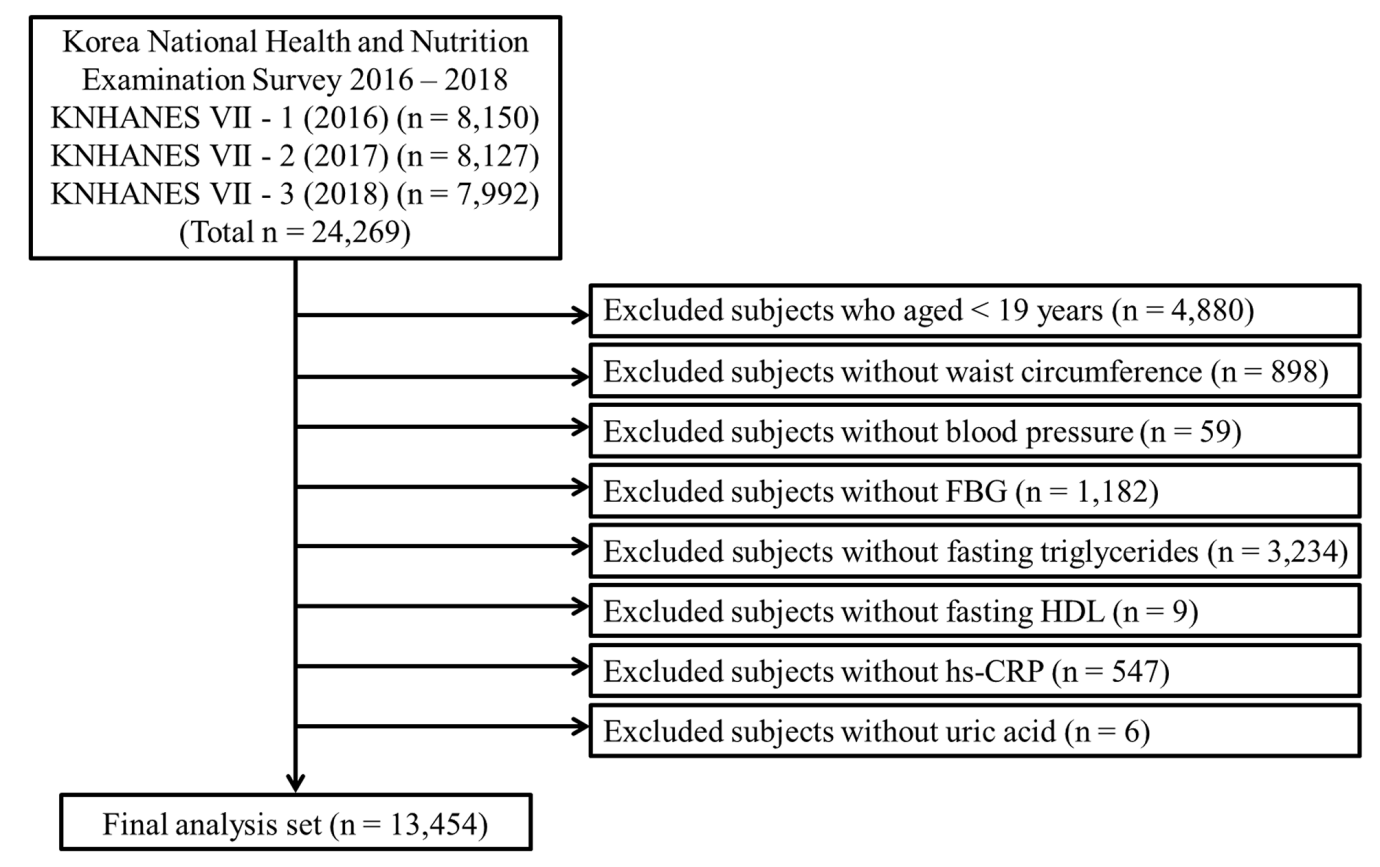
Flow chart of selecting study subjects from the Korea National Health and Nutrition Examination Survey 2016–2018

This study used data from the seventh Korea National Health and Nutrition Examination Survey (KNHANES VII) conducted during 2016–2018 by the Korean Centers for Disease Control and Prevention (KCDC). The KNHANES data were obtained annually from randomly selected 3840 individuals of 192 regions in Korea using a stratified multi-stage probability sampling design. The KNHANES included a health interview, health examination, and a nutrition survey. The data were collected through household interviews, and standardized physical examinations were conducted at mobile examination centers. All survey procedures were approved by the institutional review board of the KCDC, and written informed consent was obtained from all participants before initiating the surveys. All study participants were aged ≥ 19 years. Individuals with data on WC, BP, FBG, TG, HDL, hs-CRP, and SUA were included in our study. Finally, 13,454 out of 24,269 adults were eligible for this study (Fig. 1).

### Definition of metabolic syndrome components

Serum levels of glucose, TG, and HDL cholesterol were measured after 8- or 12-hour fasting period. The hexokinase UV method with the Hitachi Automatic Analyzer (Hitachi, Japan) and Pureauto S GLU reagent (Sekisui, Japan) was used to measure glucose levels. TG levels were measured using the enzymatic method with the Hitachi Automatic Analyzer (Hitachi, Japan) and Pureauto S TG-N reagent (Sekisui, Japan). HDL cholesterol levels were measured with the Cholestest N HDL reagent (Sekisui, Japan) and homogeneous enzymatic colorimetric method using the Hitachi Automatic Analyzer (Hitachi, Japan). Normal fasting glucose level was considered as < 100 mg/dL, impaired fasting glucose or prediabetes was considered if the fasting glucose level was 100–125 mg/dL, and DM was considered if fasting glucose was ≥ 126 mg/dL [17]. Individuals with DM who were receiving antihyperglycemic agents and/or insulin, regardless of the fasting glucose level, were also included in the DM group. Hypertriglyceridemia was defined as fasting TG level ≥ 200 mg/dL, and individuals were assigned to high or very high groups according to the National Cholesterol Education Program Adult Treatment Panel III (NCEP ATP III) guidelines [2, 18]. This decision was made to better reflect the actual clinical setting and to better capture the increased risk of cardiovascular diseases such as heart attacks and strokes. In addition, this decision was made based on the assumption that using this threshold would demonstrate a more significant association between hs-CRP, SUA, and high triglyceride levels. Low HDL cholesterol level was defined as < 40 mg/dL for men and < 50 mg/dL for women [18].

BP measurements were performed on the right arm of the participants seated for at least 5 min using a standard mercury sphygmomanometer. Three measurements were performed for all participants at 5-minute intervals, and the average of the second and third measurements was used in the analysis. Hypertension (HTN) was defined as systolic blood pressure (SBP) ≥ 140 mmHg, diastolic blood pressure (DBP) ≥ 90 mmHg, or use of antihypertensive medications independently of BP [19].

WC measurement was taken to the nearest 0.1 cm in a horizontal plane at the midpoint between the iliac crest and the lower rib. High WC was defined as ≥ 90 cm for men and ≥ 85 cm for women according to the criteria for abdominal obesity defined by the Korean Society for the Study of Obesity [20].

### Hs-CRP and SUA measurements

Hs-CRP levels were measured by immunoturbidimetry using the Cobas analyzer (Roche, Germany) and the Cardiac C-Reactive Protein High Sensitivity reagent (Roche, Germany) (detection range 0.1–20 mg/L). High and low hs-CRP levels were defined as ≥ 3.0 mg/L and < 3.0 mg/dL, respectively [1]. SUA was measured by colorimetric method using uricase and Uric acid reagent (Eiken, Japan) on a Hitachi Automatic Analyzer (Hitachi, Japan) (lower detection limit 1.0 mg/dL). High and low SUA levels were defined as ≥ 6 mg/dL and < 6 mg/dL, respectively [10].

### Other variables

Information on age, sex, socioeconomic variables (i.e., household income and educational level), and lifestyle variables (i.e., smoking, alcohol consumption, and physical activity) were collected from all participants using a self-reported questionnaire. The average monthly household income was categorized into four groups: low, lower middle, higher middle, and high. Educational level was categorized into four groups: elementary school graduation or lower, middle school graduation, high school graduation, and college graduation or higher. Non-smokers were defined as individuals who had never smoked in their lifetime, past-smokers as those who had smoked in the past but did not smoke at the time of conducting the survey, and current smokers as those who continued smoking daily or often at the time of conducting the survey. Alcohol consumption was dichotomized into zero and non-zero consumption. Physical activity during work, transport, and leisure time was estimated based on the total time spent in physical activity per week and the intensity of the physical activity. The following criteria regarding physical activity for health were recommended to achieve ≥ 600 MET-min/week: at least 150 min/week of moderate-intensity physical activity, 75 min/week of vigorous-intensity physical activity, or an equivalent combination of moderate- and vigorous-intensity physical activity.

Body weight and height were measured to the nearest 0.1 kg and 0.1 cm, respectively, with participants wearing light indoor clothing without shoes. BMI was calculated by dividing weight (in kilograms) by the square of height (in meters) (kg/m^2^). Underweight status was defined as BMI < 18.5 kg/m^2^, normoweight as BMI between ≥ 18.5 kg/m^2^ and < 25.0 kg/m^2^, overweight as BMI between ≥ 25.0 kg/m^2^ and < 30.0 kg/m^2^, and obese as BMI ≥ 30.0 kg/m^2^ [21].

### Statistical analysis

All statistical analyses were performed using SAS version 9.4 (SAS Institute Inc., Cary, NC, USA). We applied the complex sampling design and sampling weights while analyzing the KNHANES data in order to provide a national prevalence estimate. A P-value < 0.05 was considered statistically significant. The subjects included in the analysis were divided into groups based on hs-CRP and SUA levels. Subgroup analyses were performed according to sex and BMI. The chi-square test was used to analyzed categorical variables (represented as frequency and percentage (%)), whereas the independent t-test was used to analyze continuous variables (represented as mean ± standard error). Multivariate logistic regression analysis was performed to assess the impact of hs-CRP and SUA (using low hs-CRP and low SUA as the reference groups) on the prevalence of MetS components by sex and BMI (< 25.0 kg/m^2^ and ≥ 25.0 kg/m^2^) subgroups. Results of the regression analysis are presented as odds ratios (ORs) with 95% confidence intervals (CIs).

## Results

We retrospectively analysed the data of 13,454 subjects. Low and high hs-CRP levels were observed in 12,290 (91.3%) and 1,164 (8.7%) subjects, respectively. Moreover, low and high SUA levels were observed in 10,158 (75.5%) and 3,296 (24.5%) subjects, respectively. The characteristics of the participants are summarized in Table 1. Compared to subjects with low hs-CRP, the prevalence of high WC (46.16% vs. 27.69%), HTN (36.30% vs. 27.57%), prediabetes (25.14% vs. 22.87%), DM (16.10% vs. 10.18%), hypertriglyceridemia (18.18% vs. 15.48%), and low HDL (47.35% vs. 30.08%) was higher in those with high hs-CRP. Similarly, compared to subjects with low SUA, the prevalence of high WC (40.41% vs. 24.98%), HTN (33.91% vs. 26.17%), prediabetes (28.98% vs. 20.80%), hypertriglyceridemia (26.92% vs. 11.45%), and low HDL (32.10% vs. 31.31%) was higher in those with high SUA. However, the prevalence of DM was lower in subjects with high SUA compared to those with low SUA (8.87% vs. 11.36%).

**Table 1:** Characteristics of the study population

Characteristic	Low hs-CRP (N = 12,290)		High hs-CRP (N = 1,164)		p-value^*^	Low SUA (N = 10,158)		High SUA (N = 3,296)		p-value^*^
	% (SE)	Unweighted N	% (SE)	Unweighted N		% (SE)	Unweighted N	% (SE)	Unweighted N	
Age (years), mean (SE)	47.77 (0.26)	-	49.92 (0.63)	-	0.0005	49.03 (0.28)	-	44.42 (0.36)	-	< 0.0001
Gender										
Men	48.53 (0.46)	5,232	49.90 (0.21)	544	0.4459	34.12 (0.46)	3,059	86.81 (0.50)	2,717	< 0.0001
Women	51.47 (0.45)	7,058	50.01 (0.19)	620		65.88 (0.45)	7,099	13.19 (0.18)	579	
Household income										
Low	16.04 (0.52)	2,387	23.61 (0.14)	337	< 0.0001	17.55 (0.50)	2,117	14.36 (0.23)	607	0.0001
Lower middle	23.66 (0.57)	2,988	24.60 (0.14)	287		23.97 (0.50)	2,485	23.12 (0.31)	790	
Higher middle	29.46 (0.61)	3,379	29.05 (0.17)	289		29.50 (0.52)	2,779	29.22 (0.33)	889	
High	30.84 (0.82)	3,500	22.75 (0.14)	245		28.98 (0.68)	2,744	33.30 (0.39)	1,001	
Educational level										
≤ Elementary school	15.08 (0.46)	2,505	20.07 (0.12)	319	0.0004	17.52 (0.45)	2,305	10.14 (0.15)	519	< 0.0001
Middle school	8.68 (0.30)	1,193	9.67 (0.09)	115		9.27 (0.29)	1,004	7.45 (0.14)	304	
High school	34.42 (0.61)	3,731	32.16 (0.17)	309		33.74 (0.56)	3,022	35.51 (0.34)	1,018	
≥ College	41.82 (0.81)	4,446	38.10 (0.18)	373		39.47 (0.63)	3,477	46.90 (0.45)	1,342	
Smoking										
Non-smoker	58.21 (0.51)	7,514	55.84 (0.21)	652	0.0476	66.64 (0.50)	6,994	35.37 (0.33)	1,172	< 0.0001
Ex-smoker	21.64 (0.39)	2,627	20.34 (0.13)	260		17.85 (0.33)	1,779	31.19 (0.29)	1,108	
Current smoker	20.15 (0.48)	2,067	23.82 (0.14)	235		15.51 (0.37)	1,306	33.44 (0.35)	996	
Alcohol consumption										
No	8.92 (0.30)	1,386	11.24 (0.10)	148	0.0296	10.80 (0.29)	1,339	4.69 (0.12)	195	< 0.0001
Yes	91.08 (0.40)	10,830	88.76 (0.26)	1,002		89.20 (0.53)	8,749	95.31 (0.50)	3,083	
BMI (kg/m^2^)										
< 25	65.99 (0.56)	8,079	48.30 (0.19)	583	< 0.0001	70.27 (0.54)	7,009	49.39 (0.40)	1,653	< 0.0001
≥ 25	34.01 (0.52)	4,180	51.70 (0.21)	579		29.73 (0.48)	3,121	50.61 (0.40)	1,638	
High waist circumference	27.69 (0.52)	3,617	46.16 (0.19)	544	< 0.0001	24.98 (0.45)	2,761	40.41 (0.35)	1,400	< 0.0001
Hypertension	27.57 (0.52)	4,006	36.30 (0.16)	485	< 0.0001	26.17 (0.47)	3,170	33.91 (0.30)	1,321	< 0.0001
Glucose metabolism status										
Normal	66.95 (0.56)	7,807	58.76 (0.22)	621	< 0.0001	67.84 (0.54)	6,573	62.15 (0.45)	1,855	< 0.0001
Prediabetes	22.87 (0.40)	2,959	25.14 (0.14)	326		20.80 (0.37)	2,244	28.98 (0.28)	1,041	
DM	10.18 (0.31)	1,524	16.10 (0.12)	217		11.36 (0.29)	1,341	8.87 (0.15)	400	
Hypertriglyceridemia	15.48 (0.36)	1,824	18.18 (0.13)	196	0.0489	11.45 (0.30)	1,138	26.92 (0.27)	882	< 0.0001
Low HDL cholesterol	30.08 (0.51)	4,027	47.35 (0.20)	573	< 0.0001	31.31 (0.45)	3,448	32.10 (0.30)	1,152	0.4404
Physical activity										
< 600 MET-minutes/week	53.13 (0.60)	6,692	56.31 (0.21)	669	0.0835	54.85 (0.58)	5,673	49.55 (0.39)	1,688	< 0.0001
≥ 600 MET-minutes/week	46.87 (0.62)	5,174	43.69 (0.19)	448		45.15 (0.54)	4,133	50.45 (0.40)	1,489	
Number of MetS components										
< 3	71.71 (0.55)	8,379	57.41 (0.22)	616	< 0.0001	74.07 (0.56)	7,146	61.17 (0.45)	1,849	< 0.0001
≥ 3	28.29 (0.50)	3,911	42.59 (0.17)	548		25.93 (0.45)	3,012	38.83 (0.32)	1,447	

hs-CRP, high sensitivity C-reactive protein; SUA, serum uric acid; SE, standard error; BMI, body mass index; DM, diabetes mellitus; HDL, high density lipoprotein; MET, metabolic equivalents; MetS, metabolic syndrome

^*^Calculated by chi-square tests for categorical variables and independent t-tests for continuous variables

To determine the association between hs-CRP levels and MetS components, we performed logistic regression analysis, and the results are presented in Table 2. A significant negative correlation was observed between HDL and hs-CRP (OR, 1.703; 95% CI, 1.431–2.027). In subjects with BMI < 25 kg/m^2^, the OR of high hs-CRP for low HDL was higher than that for low hs-CRP. In subjects with BMI ≥ 25 kg/m^2^, the OR of high hs-CRP as a risk determinant factor for low HDL was higher than that of low hs-CRP. However, the OR in subjects with BMI < 25 kg/m^2^ was relatively higher than that in their counterparts. High WC was significantly associated with hs-CRP only in subjects with BMI ≥ 25 kg/m^2^. When stratified by sex, similar trends were observed.

**Table 2:** Odds ratios for the components of the metabolic syndrome according to hs-CRP levels

Metabolic syndrome components	Unadjusted OR (95% CI)	Adjusted OR (95% CI)	Adjusted OR (95% CI) for BMI < 25 kg/m^2a^	Adjusted OR (95% CI) for BMI ≥ 25 kg/m^2a^
Total				
HTN^b^	1.497 (1.296, 1.730)	1.001 (0.835, 1.200)	1.095 (0.854, 1.402)	1.168 (0.916, 1.489)
DM^c^	1.802 (1.470, 2.208)	1.091 (0.855, 1.392)	0.991 (0.704, 1.396)	1.314 (0.949, 1.819)
Prediabetes^c^	1.253 (1.055, 1.488)	0.923 (0.766, 1.111)	0.994 (0.765, 1.292)	0.961 (0.746, 1.238)
Hypertriglyceridemia^d^	1.213 (1.000, 1.471)	0.772 (0.622, 0.958)	0.805 (0.577, 1.124)	0.782 (0.595, 1.028)
Low HDL^e^	2.090 (1.791, 2.440)	1.703 (1.431, 2.027)	1.943 (1.532, 2.464)	1.571 (1.228, 2.011)
High waist circumference^f^	2.239 (1.946, 2.576)	0.989 (0.799, 1.223)	1.066 (0.741, 1.534)	1.653 (1.243, 2.199)
Men^*^				
HTN^b^	1.305 (1.064–1.600)	0.931 (0.726–1.193)	0.891 (0.636–1.247)	1.149 (0.817–1.616)
DM^c^	1.576 (1.171–2.121)	1.099 (0.785–1.538)	0.930 (0.582–1.486)	1.403 (0.871–2.258)
Prediabetes^c^	1.186 (0.929–1.516)	0.938 (0.727–1.210)	1.102 (0.752–1.616)	0.890 (0.620–1.276)
Hypertriglyceridemia^d^	0.879 (0.672–1.148)	0.622 (0.470–0.824)	0.654 (0.419–1.020)	0.611 (0.433–0.862)
Low HDL^e^	1.863 (1.497–2.318)	1.557 (1.235–1.962)	1.709 (1.215–2.403)	1.532 (1.090–2.155)
High waist circumference^f^	1.794 (1.432–2.248)	1.054 (0.774–1.436)	1.128 (0.650–1.957)	1.764 (1.139–2.732)
Women^*^				
HTN^b^	1.724 (1.414–2.102)	1.128 (0.862–1.477)	1.401 (0.977–2.007)	1.182 (0.835–1.674)
DM^c^	2.066 (1.573–2.712)	1.050 (0.748–1.474)	1.063 (0.635–1.780)	1.198 (0.795–1.803)
Prediabetes^c^	1.316 (1.044–1.660)	0.897 (0.694–1.159)	0.841 (0.593–1.192)	1.052 (0.742–1.491)
Hypertriglyceridemia^d^	1.939 (1.463–2.569)	1.136 (0.821–1.573)	1.191 (0.723–1.962)	1.100 (0.737–1.640)
Low HDL^e^	2.432 (1.971–3.000)	1.832 (1.442–2.326)	2.114 (1.545–2.892)	1.632 (1.164–2.288)
High waist circumference^f^	2.814 (2.328–3.401)	0.977 (0.725–1.316)	1.014 (0.627–1.642)	1.599 (1.092–2.342)

Odds ratios with adjustments using logistic regression models

HTN, hypertension; DM, diabetes mellitus

^*^Not adjusted for sex

^a^Not adjusted for BMI.

^b^Adjusted for age, sex, household income, educational level, smoking, alcohol consumption, BMI (continuous), DM, prediabetes, hypertriglyceridemia, low HDL, high waist circumference, and physical activity

^c^Adjusted for age, sex, household income, educational level, smoking, alcohol consumption, BMI (continuous), hypertension, hypertriglyceridemia, low HDL, high waist circumference, and physical activity

^d^Adjusted for age, sex, household income, educational level, smoking, alcohol consumption, BMI (continuous), hypertension, DM, prediabetes, low HDL, high waist circumference, and physical activity

^e^Adjusted for age, sex, household income, educational level, smoking, alcohol consumption, BMI (continuous), hypertension, DM, prediabetes, hypertriglyceridemia, high waist circumference, and physical activity

^f^Adjusted for age, sex, household income, educational level, smoking, alcohol consumption, BMI (continuous), hypertension, DM, prediabetes, hypertriglyceridemia, low HDL, and physical activity

Furthermore, logistic regression analysis was performed to determine the association between SUA levels and MetS components, and the results are summarized in Table 3. A high SUA level was found to be an independent risk factor for HTN (OR, 1.399; 95% CI, 1.210–1.616), hypertriglyceridemia (OR, 1.735; 95% CI, 1.486–2.026), and low HDL (OR, 1.257; 95% CI, 1.106–1.429), but not for DM (OR, 0.478; 95% CI, 0.382–0.597). The ORs of high SUA for prediabetes and high WC were similar to those for low SUA. Similar trends were observed when the subjects were stratified by BMI (< 25 kg/m^2^ vs. ≥25 kg/m^2^).

**Table 3:** Odds ratios for the components of the metabolic syndrome according to uric acid levels

Metabolic syndrome components	Unadjusted OR (95% CI)	Adjusted OR (95% CI)	Adjusted OR (95% CI) for BMI < 25 kg/m^2a^	Adjusted OR (95% CI) for BMI ≥ 25 kg/m^2a^
Total				
HTN^b^	1.448 (1.307–1.605)	1.399 (1.210–1.616)	1.479 (1.222–1.790)	1.478 (1.235–1.769)
DM^c^	0.852 (0.734–0.989)	0.478 (0.382–0.597)	0.519 (0.389–0.694)	0.499 (0.372–0.669)
Prediabetes^c^	1.521 (1.369–1.690)	0.937 (0.817–1.074)	0.927 (0.765–1.123)	0.995 (0.822–1.204)
Hypertriglyceridemia^d^	2.849 (2.534–3.203)	1.735 (1.486–2.026)	1.920 (1.536–2.399)	1.579 (1.293–1.927)
Low HDL^e^	1.037 (0.945–1.139)	1.257 (1.106–1.429)	1.328 (1.115–1.581)	1.212 (1.010–1.456)
High waist circumference^f^	2.038 (1.856–2.237)	1.042 (0.887–1.225)	1.233 (0.921–1.650)	1.298 (1.083–1.556)
Men^*^				
HTN^b^	0.989 (0.866–1.128)	1.272 (1.081–1.497)	1.292 (1.050–1.589)	1.402 (1.118–1.757)
DM^c^	0.384 (0.318–0.465)	0.339 (0.268–0.429)	0.396 (0.291–0.537)	0.326 (0.232–0.458)
Prediabetes^c^	0.953 (0.834–1.088)	0.845 (0.725–0.984)	0.866 (0.703–1.066)	0.858 (0.676–1.088)
Hypertriglyceridemia^d^	1.879 (1.611–2.191)	1.735 (1.452–2.074)	1.889 (1.490–2.393)	1.611 (1.258–2.062)
Low HDL^e^	1.351 (1.183–1.542)	1.171 (1.002–1.368)	1.247 (1.016–1.532)	1.159 (0.915–1.467)
High waist circumference^f^	1.586 (1.396–1.801)	0.933 (0.770–1.132)	1.055 (0.756–1.471)	1.203 (0.954–1.517)
Women^*^				
HTN^b^	3.225 (2.613–3.981)	1.906 (1.443–2.517)	2.365 (1.631–3.430)	1.783 (1.291–2.461)
DM^c^	3.405 (2.583–4.489)	1.512 (1.081–2.114)	1.880 (1.176–3.004)	1.425 (0.958–2.120)
Prediabetes^c^	2.606 (2.070–3.281)	1.431 (1.100–1.861)	1.404 (0.953–2.070)	1.459 (1.035–2.057)
Hypertriglyceridemia^d^	3.193 (2.478–4.115)	1.752 (1.331–2.305)	2.151 (1.409–3.283)	1.522 (1.082–2.142)
Low HDL^e^	2.441 (2.009–2.967)	1.483 (1.189–1.850)	1.454 (1.058–1.998)	1.428 (1.057–1.930)
High waist circumference^f^	3.900 (3.180–4.783)	1.598 (1.145–2.230)	1.697 (1.070–2.693)	1.731 (1.209–2.478)

Odds ratios with adjustments using logistic regression models

HTN, hypertension; DM, diabetes mellitus

^*^Not adjusted for sex

^a^Not adjusted for BMI.

^b^Adjusted for age, sex, household income, educational level, smoking, alcohol consumption, BMI (continuous), DM, prediabetes, hypertriglyceridemia, low HDL, high waist circumference, and physical activity

^c^Adjusted for age, sex, household income, educational level, smoking, alcohol consumption, BMI (continuous), hypertension, hypertriglyceridemia, low HDL, high waist circumference, and physical activity

^d^Adjusted for age, sex, household income, educational level, smoking, alcohol consumption, BMI (continuous), hypertension, DM, prediabetes, low HDL, high waist circumference, and physical activity

^e^Adjusted for age, sex, household income, educational level, smoking, alcohol consumption, BMI (continuous), hypertension, DM, prediabetes, hypertriglyceridemia, high waist circumference, and physical activity

^f^Adjusted for age, sex, household income, educational level, smoking, alcohol consumption, BMI (continuous), hypertension, DM, prediabetes, hypertriglyceridemia, low HDL, and physical activity

Finally, logistic regression analysis was implemented to estimate the association between SUA levels and MetS components for men and women separately, and the findings are presented in Table 3. In the subgroup analyses for men, the ORs of high SUA for HTN (OR, 1.272; 95% CI, 1.081–1.497), hypertriglyceridemia (OR, 1.735; 95% CI, 1.452–2.074), and low HDL (OR, 1.171; 95% CI, 1.002–1.368) were higher than those of low SUA. However, the ORs of high SUA for DM (OR, 0.339; 95% CI, 0.268–0.429) and prediabetes (OR, 0.845; 95% CI, 0.725–0.984) were lower than those of low SUA. No significant association was observed between SUA levels and WC. In men, these trends remained even after stratification according to BMI. In the subgroup analyses for women, the association between SUA and each MetS component was comparatively stronger than that in men. The ORs of high SUA for HTN (OR, 1.906; 95% CI, 1.443–2.517), DM (OR, 1.512; 95% CI, 1.081–2.114), prediabetes (OR, 1.431; 95% CI, 1.100-1.861), hypertriglyceridemia (OR, 1.752; 95% CI, 1.331–2.305), low HDL (OR, 1.483; 95% CI, 1.189–1.850), and high WC (OR, 1.598; 95% CI, 1.145–2.230) were higher than those of low SUA. In particular, a significant association between high SUA levels and DM risk was observed only among women.

## Discussion

Herein, we investigated the association of hs-CRP and SUA levels with MetS components in Korean adults aged 19 years and above using the reliable and representative KNHANES VII data. Our results showed that low HDL was more prevalent in subjects with high hs-CRP levels. This trend remained even when individuals were grouped by sex and BMI; however, the association was stronger in women than in men. Moreover, the prevalence of high SUA was higher in subjects with HTN, hypertriglyceridemia, and low HDL; however, it was lower in those with DM. When grouped by sex, this trend was still observed in men, but all MetS components were found to be more prevalent in women with high SUA. These results suggest that hs-CRP and SUA could be used as potential biomarkers for identifying individuals at high risk for MetS and cardiometabolic diseases.

Increased hs-CRP levels in individuals with possible MetS could be explained based on the prevalence of low HDL and high WC [22–25]. The inverse relationship between hs-CRP and HDL levels in healthy individuals and those with MetS has been reported in several studies, suggesting that low HDL could be the main independent determinant for high hs-CRP that favors inflammation [22, 24, 25]. Another study reported that the overall prevalence of low HDL was approximately 1.703 times higher in subjects with high hs-CRP than those with low hs-CRP. Interestingly, higher prevalence of high hs-CRP was observed in women than in men. This sex-associated difference might be due to differences in the hormonal profile and total body adipose tissue between men and women [22, 26]. In women, estrogen might be involved in the inflammatory process [22], and compared to men, women generally have a higher percentage of total body adipose tissue, which is known to secretes several proinflammatory cytokines, such as tumor necrosis factor (TNF)-α, leptin, and adiponectin [26, 27]. Additionally, previous studies have shown that CRP gene expression and levels are increased from adipose tissue of MetS subjects, suggesting the significant association between increased hs-CRP levels and high WC [28, 29].

In this study, compared to overweight and obese subjects (BMI ≥ 25 kg/m^2^), normal-weight subjects (BMI < 25 kg/m^2^) showed a more significant negative correlation between HDL and hs-CRP in both men and women. This might be explained partially by the fact that individuals with normal weight obesity (NWO), which is defined as a state of normal weight (BMI < 25 kg/m^2^) but increased body fat percentage, are at a high risk for cardiovascular disorders [30]. The NWO phenotype is more prevalent in Asians compared to Westerners [31, 32]. Previous studies showed that Asians not only have a high percentage of body fat but also a relatively high prevalence of at least one risk factor for cardiovascular disorders even at a low BMI [32]. Specifically, over 30% of the general Korean population might be classified as having NWO even if a lower cut-off (BMI < 23 kg/m^2^ for the Asian cut-off point) is applied [33]. Individuals with the NWO phenotype are likely to have increased levels of adipocyte-derived proinflammatory cytokines due to increased body fat mass [30, 34]. In people with NWO, excessive body fat could affect the levels of HDL and TG through various mechanisms, such as alteration of the lipolysis process [30, 35]. Free fatty acid (FFA) and very low-density lipoprotein-triglyceride (VLDL-TG) complex are synthesized by the adipose tissue, and increased lysis of TG leads to increased levels of FFA, which eventually prevents the action of the enzyme lipoprotein lipase and increases the production of VLDL and TG in the liver [30, 36]. Increase in FFA, TG, and VLDL-TG levels can further promote the activity of cholesteryl ester transfer protein, leading to increased synthesis of TG-rich HDL particles, which are easily cleared and degraded, thereby lowering HDL levels [30, 36].

Our results showed that the overall prevalence rate of high SUA was higher in subjects with hypertriglyceridemia but not in those with high WC, a surrogate marker of central obesity. Our results are consistent with those of previous studies [37, 38]. A cross-sectional study in Peru reported that high SUA levels are positively associated with hypertriglyceridemia but not with central obesity [37]. Another longitudinal study in China reported that individuals with higher triglyceride levels are more likely to develop hyperuricemia than those with low triglyceride levels [38]. The association between high SUA and hypertriglyceridemia can be indirectly proved by urate-lowering therapy, which improves serum cholesterol and triglyceride levels [39]. Moreover, this association between high SUA and hypertriglyceridemia could be explained to some extent by alterations in the metabolism of lipids, especially triglycerides, through the activation of mitochondrial NADPH oxidase and inhibition of AMPK and AKT2 [37, 40]. Thus, although central obesity is a significant risk factor for the development of cardiometabolic diseases and MetS, our results suggest that hypertriglyceridemia due to high SUA precedes obesity. Interestingly, differences in the sex-specific association between high SUA levels and hypertriglyceridemia were not observed in this study.

Consistent with previous studies, we observed significantly higher SUA levels in men than in women, which might be due to the urate-lowering effects of estrogen in women [41–43]. However, it has been shown that the association between high SUA and various diseases including HTN is stronger in women than in men, which suggests that SUA plays a sex-specific role in the progression of cardiometabolic diseases [41, 44]. This sensitivity of women towards SUA may be partially explained by the positive association between mean platelet volume and SUA levels, which is observed only in women [41, 45]. Mean platelet volume as a determinant of platelet activation and is correlated with several cardiometabolic diseases including HTN [41, 46–48]. We observed that the overall prevalence rate of high SUA is higher in subjects with HTN. Specifically, the relationship between high SUA and HTN was found to be stronger in women than in men. These results are contrary with those of previous studies. A study from Japan reported that high SUA is strongly associated with HTN in men, but not in women [49]. Conversely, another study from China found that high SUA is positively correlated with HTN in men, whereas a negative correlation was observed in women [50].

We observed that the overall prevalence of high SUA is lower in diabetic subjects, which is in line with the results of previous studies [51–54]. It has been reported that SUA levels are inversely correlated with DM in a representative sample of US adults [51]. This inverse correlation between SUA and DM was also reported in studies from Japan, India, and China [52–54]. This inverse correlation might be due to the uricosuric effect of glucose on uric acid. High glucose levels in diabetic individuals may prevent uric acid from being reabsorbed and enhance its excretion through the kidneys, thereby reducing the net concentration of SUA [55–57]. Another possible mechanism to explain the negative correlation between SUA and DM is related to the reduced antioxidant action of uric acid due to oxidative stress and increased free radical production in diabetic individuals [58–60]. It is possible that serum uric acid has a dual function in determining health outcomes, with the ultimate effects depending on the balance between these two functions [61]. At lower levels, serum uric acid can act as a powerful endogenous antioxidant with beneficial anti-oxidative effects [62, 63]. However, at higher levels, serum uric acid can act as a prooxidant on vascular cells and adipocytes [64], and promote the secretion of inflammatory mediators such as C-reactive protein and monocyte chemoattractant protein-1 [65, 66]. This suggests that at higher levels, its prooxidant function may outweigh its antioxidant function, leading to endocrine disorders.

Interestingly, when stratified by sex, the negative association between SUA and DM became stronger in men; however, a positive association was observed in women, which is similar to findings of some previous studies [67, 68]. A 5-year retrospective cohort study conducted in Korea reported a significant association between hyperuricemia and the risk for DM only in women but not in men [67]. Another retrospective longitudinal study conducted in Japan also reported similar association only in women [68]. These sex-associated differences might be to some extent due to differences in fat distribution and its role in regulating SUA levels between men and women. In women, low glucocorticoid receptor gene expression and low glucocorticoid-binding were observed in visceral preadipocytes compared to subcutaneous preadipocytes, which may lead to decreased visceral fat mass in women [69]. It has also been reported that subcutaneous-fat-type and visceral-fat-type obesity may be implicated in poor uric acid excretion and excess uric acid production, respectively [70]. Moreover, changes in the transport of uric acid in the kidney tubules may lead to insulin resistance or hyperinsulinemia in individuals with reduced uric acid excretion [71].

Our study has some limitations which have be considered when interpreting the results. The main limitation of our study is the cross-sectional study design, which makes it difficult to clearly determine the causality between hs-CRP, SUA, and MetS components. Second, almost all variables measured at a single time point were used to identify the effects of hs-CRP and SUA on the prevalence of each MetS component, which might negatively affect the data accuracy. Third, recall bias while collecting the sociodemographic characteristics cannot be ruled out due to the use of surveys. Finally, the overall prevalence of MetS components might have been underestimated since we included subjects without complete information on them. However, the data could be missing at random; thus, the exclusion process is unlikely to significantly affect the study findings.

## Conclusion

Low HDL is more prevalent in individuals with high hs-CRP levels, and this association between HDL and hs-CRP is stronger in women than in men. Moreover, the prevalence of high SUA is more in people with HTN, hypertriglyceridemia, and low HDL; however, it is less in diabetic individuals. When grouped by sex, this trend is still observed in men, but all MetS components are more prevalent in women with high SUA.

## Data Availability

The datasets used for this study are available from the corresponding author on reasonable request.
